# The Gut Viral Metagenome Analysis of Domestic Dogs Captures Snapshot of Viral Diversity and Potential Risk of Coronavirus

**DOI:** 10.3389/fvets.2021.695088

**Published:** 2021-07-07

**Authors:** Ying Shi, Jie Tao, Benqiang Li, Xiaohui Shen, Jinghua Cheng, Huili Liu

**Affiliations:** ^1^Department of Animal Infectious Diseases, Institute of Animal Husbandry and Veterinary Sciences, Shanghai Academy of Agricultural Sciences, Shanghai, China; ^2^Shanghai Key Laboratory of Agricultural Genetic Breeding, Shanghai, China; ^3^Shanghai Engineering Research Center of Pig Breeding, Shanghai, China

**Keywords:** domestic dogs, gut virome, viral metagenomics, public health, coronavirus

## Abstract

The close relations between dogs (Canis lupus familiaris) and humans lay a foundation for cross species transmissions of viruses. The co-existence of multiplex viruses in the host accelerate viral variations. For effective prediction and prevention of potential epidemic or even pandemic, the metagenomics method was used to investigate the gut virome status of 45 domestic healthy dogs which have extensive contact with human beings. A total of 248.6 GB data (505, 203, 006 valid reads, 150 bp in length) were generated and 325, 339 contigs, which were best matched with viral genes, were assembled from 46, 832, 838 reads. In the aggregate, 9,834 contigs (3.02%) were confirmed for viruses. The top 30 contigs with the most reads abundance were mapped to DNA virus families Circoviridae, Parvoviridae and Herpesviridae; and RNA virus families Astroviridae, Coronaviridae and Picornaviridae, respectively. Numerous sequences were assigned to animal virus families of Astroviridae, Coronaviridae, Circoviridae, etc.; and phage families of Microviridae, Siphoviridae, Ackermannviridae, Podoviridae, Myoviridae and the unclassified phages. Further, several sequences were homologous with the insect and plant viruses, which reflects the diet and habitation of dogs. Significantly, canine coronavirus was uniquely identified in all the samples with high abundance, and the phylogenetic analysis therefore showed close relationship with the human coronavirus strain 229E and NL63, indicating the potential risk of canine coronavirus to infect humans by obtaining the ability of cross-species transmission. This study emphasizes the high detection frequency of virus harbored in the enteric tract of healthy contacted animal, and expands the knowledge of the viral diversity and the spectrum for further disease-association studies, which is meaningful for elucidating the epidemiological and biological role of companion animals in public health.

## Introduction

Dogs (Canis lupus familiaris), one of the most popular companion animals, have extensive interactions with humans through sharing spaces, occasionally biting and scratching, playing, and producing fecal waste. Besides, dogs also frequently hunt or scavenge wildlife, increasing the potential transmission risk of zoonosis, as exemplified by rotavirus and rabies virus (1–3).

Domestic dogs are the potential source of zoonosis for human during the direct contact with infected dogs or indirect contact via dog feces (4). The infectious or zoonotic pathogens in infected dogs might transfer to human and cause infection after inadvertent ingestion. This kind of infection caused by canine fecal zoonoses is always ignored or misdiagnosed due to the non-specific clinical signs or the unaware of the presence of the zoonotic pathogens. Even though dogs are getting popular as pets in the city and the potential zoonotic risk from the close contact between human and dogs, few contemporary studies were carried out to characterize the viral content of canine feces. Till now, there are several viral infections identified affecting the health of dogs (5–7). Canine distemper virus (8) and canine parvovirus2 (CPV2) (9, 10) are distributed globally and highly contagious. Other viruses such as canine rotavirus (11), adenovirus, herpesvirus (12), influenza virus (13), papillomavirus (14) and parainfluenza virus (15) have also been reported as potential dog pathogens. Canine coronavirus (CCoV) including enteric CCoVs, canine respiratory coronavirus and canine pantrophic coronavirus were identified in previous research (16–18). A better understood of the zoonotic pathogens and the health risk posed by domestic dogs would prompt to institute effective strategies more efficiently in the prevention of human infections.

Thanks to the development and application of next generation sequencing (NGS) technology in viral metagenomics, it has been practical for large-scale detection of known and unknown viruses in the reservoir hosts (19, 20), including human, turkey, pig, cow, bat, cat, horse, chicken, rodents, pigeon, duck, ferrets and other animals (21–32). Several novel or uncommon virus strains were identified and isolated successfully with this approach. A clear virome status in the reservoir hosts is also necessary to control the outbreaks of viral diseases and to prevent the transmission (33). However, for domestic dogs in the city, more information and research were in need for the viral diversity in the enteric tract to achieve knowledge of potential enteric pathogen risk and a better diagnose of the diseases.

In this research, metagenome was applied to obtain an unbiased measure of the viral diversity in the guts of domestic dogs. The fecal viruses in specimens from sheltered and pet dogs were characterized, suggesting a wide range of viral sequences related to plant, animal, insect, and phages. This viral genome information from the enteric tract of healthy domestic dogs presented a baseline for fecal virome and is a good reference for future identification of composition changes, which may indicate the potential risk for public health security.

## Materials and Methods

### Ethics Statement

All animal experiments followed the recommendations in the Guide for the Care and Use of Laboratory Animals of the Ministry of Science and Technology of the People's Republic of China. The Animal Care and Use Committee of Shanghai Academy of Agricultural Science reviewed protocols, including operation details as well as approaches to ameliorate animal suffering and euthanasia.

### Specimens and Pretreatment

A total of 45 fecal specimens of domestic dogs were collected from five pet hospitals located in four urban districts (Jiading, Minhang, Songjiang and Jing'an), in which the communities have large populations more than 10,000. Aiming to better reflect the conventional status of gut virome in domestic dogs, the dogs were randomly selected with the criteria of being healthy and behaving normally, and the vaccination situation was recorded. The fecal specimen was placed in the sterile-tipped eppendorf tube and frozen at −80 °C within 4–8 h of collection. One g of the fecal specimen was washed with 10 volumes of precooled sterile SB buffer (0.2 M NaCl, 5 mM CaCl_2_, 50 mM Tris-HCl, 5mM MgCl_2_, pH 7.5) after three rounds of freezing-thawing. The samples were centrifuged at 4°C for 5 min at 1,000 ×g, 3,000 ×g, 5,000 ×g, 8,000 ×g, 10,000 ×g and 12,000 ×g separately to remove the precipitate. After precooling on ice for10 min, the cell fragments were removed by 0.22 μm ultrafiltration tube and the supernatant was transferred to the ultracentrifugation tube containing 28% (w/W) sucrose to centrifuge at 300,000 ×g for 2 h using HIMAC CP 100 wx ultracentrifuge (Hitachi, Tokyo, Japan). The precipitate was suspended in buffer solution (90 uL 10× DNase I Buffer, 90 uL 1 U/uL DNase I, 0.9 uL 100 mg/mL RNaseA, 720 uL sterile water) and shook for 60 min at 37°C to digest non-particle-protected nucleic acids, then stored at 4°C overnight.

### Extraction of Viral Nucleic Acids

DNA and RNA were co-extracted from the pretreatment samples with MagPure Viral DNA/RNA Mini LQ Kit (Magen kitR6662-02), while the equivalent SB buffer was used as a blank control. The viral RNA was reverse transcribed according to the specification of SuperScript III reverse transcriptase (Invitrogen) with random hexamer primer. Afterwords, SMARTer Ultra Low input RNA kit was used to synthesize double strand cDNA. The double-stranded DNA fragments comprising of 3′ or 5′ overhangs were generated and end-repaired followed by A-tail adapter ligation. The whole genome was amplified with Qiagen kit according to manufacturer's instructions. The viral nucleic acids were quantified by the NanoDrop spectrophotometer (Thermo Fisher Scientific) and 1.5% agarose electrophoresis.

### Library Generation and Sequencing

Following manufacturer's instructions, the Next® Ultra™DNA Library Prep Kit for Illumina® (New England Biolabs) was used to generate the sequencing libraries and add the index codes. The library quality was assessed by the Qubit® dsDNA HS Assay Kit (Life Technologies) and Agilent 4,200 system (Agilent, Santa Clara). High-throughput sequencing was conducted on an Illumina Novaseq 6,000 and 150 bp paired-end reads were generated by the Magigene Company (Guangzhou, China). Eighteen libraries were then constructed (Supplementary Table 1).

### Data Analysis

The raw sequencing reads were processed to acquire the clean data using Soapnuke (v2.0.5) (34) for further analysis. The sliding-window algorithm was used to trim the reads with low quality after removing the adapters from sequencing reads using Cutadapt (v1.2.1). All clean reads were mapped to the host reference genome of Canis lupus familiaris (dog) and the ribosomal database (silva.132) (35) utilizing BWA (v0.7.17) (36) to avoid the confusion caused by host sequences and ribosomes. The obtained quality-filtered reads were then de novo assembled to generate the metagenome for each sample. Clean reads without host sequences and ribosomes were mapped to the GenBank non-redundant nucleotide (NT) database to identify virus reads primarily. Different virus families were classified according to the annotation information from NCBI taxonomy database.

### Phylogenetic Analysis

Nucleotide sequences (https://www.ncbi.nlm.nih.gov/sra/PRJNA734701) were firstly assembled from contigs and then aligned using CLUSTALW with the default settings (37). Aligned sequences were trimmed to match the genomic regions of the viral sequences obtained in the study. The reference sequences, including dog viral sequences, related viral species or genera and the best BLASTp hits, were obtained from the GenBank database. Phylogenetic trees were constructed using the neighbor joining method by MEGA 6.06 (38) with 1,000 bootstrap replicates.

## Results

### Viral Metagenomics

A total of 45 fecal specimens of domestic dogs from five pet hospitals were used to reveal viral diversity of urban domestic dogs. Five samples were mixed to nominate a same ID for deep sequence of both DNA and RNA database, therefore; a total of 18 data IDs were generated (Supplementary Table 1). A total of 248.6 GB NGS data related to viral nucleic acids sequences were obtained from platform Illumina containing 505,203,006 valid reads (150 bp in length). Hereinto, there were 46,832,838 reads (~0.09% of the total sequence reads) were best matched with viral proteins available in the NCBI database. In each sample, the number of reads associated to virus varied from 16,907 to 16,859,924 (Supplementary Table 1). In the aggregate, 31 DNA and 32 RNA families of viruses were parsed. MEGAHIT (version 1.0) (39) was utilized to generate 325,339 unique contigs with a max length of 643,829 bp. A total of 9,834 contigs (3.02%) were assigned for virus species, in taxonomic assignment on the basis of BLAST analysis (Supplementary Table 2).

**Figure 1:**
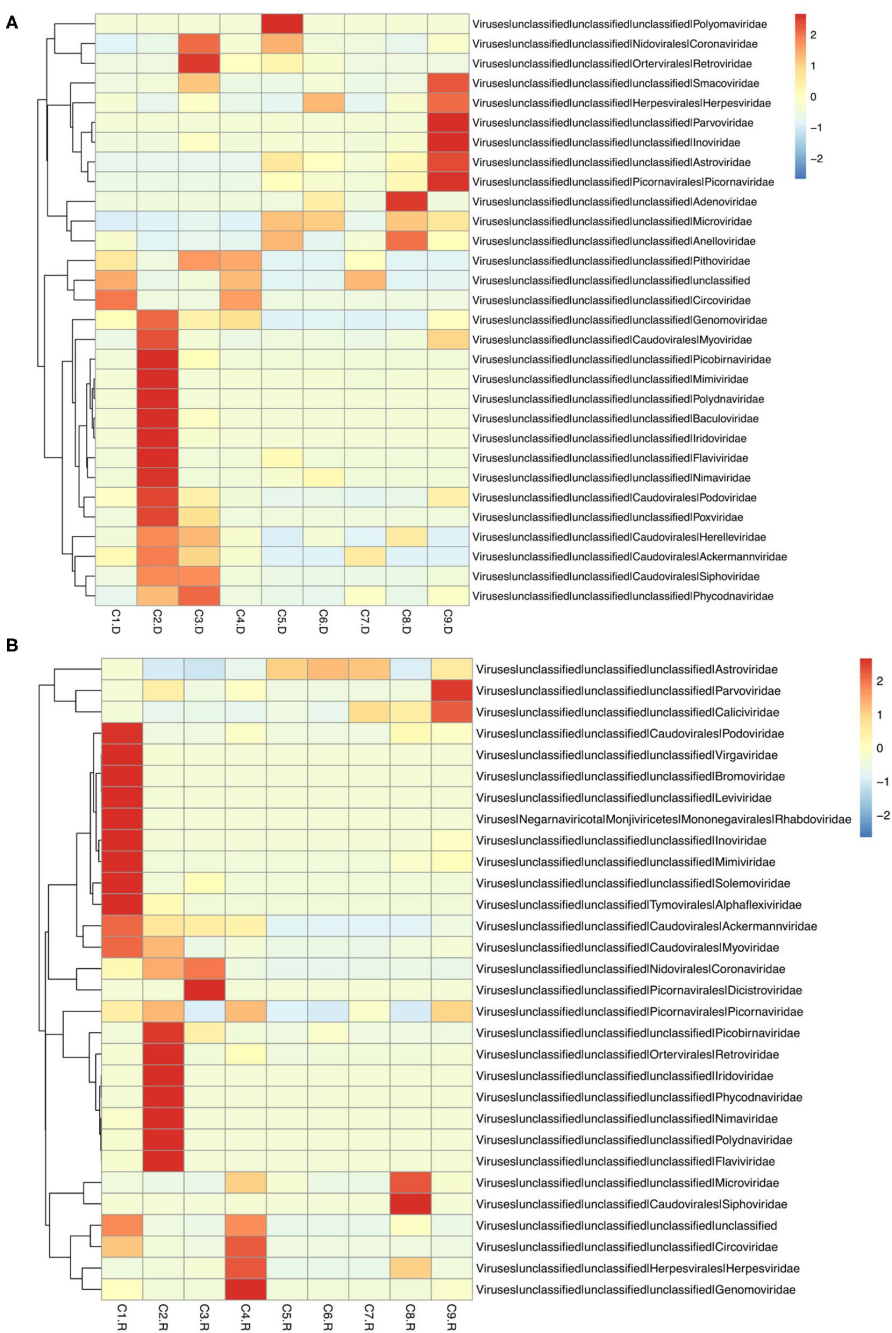
Heatmap of contigs with the top 30 abundance of sequence reads in each sample. **(A)** The heatmap of DNA virus contigs. **(B)** The heatmap of RNA virus contigs. Information of contigs and the virus families was provided in the right text column. The samples were listed below the heatmap. The boxes colored from blue to red represent the abundance of virus reads aligned to each contig.

Different types of viral genomes were identified, including 87.63% DNA viruses and 12.37% RNA viruses. And 78.35% of the RNA viruses were assigned to be Phages. There were also 42,359 contigs suspected to virus species, 91.45% accounting for DNA viruses, 8.55% for RNA viruses, and 76.12% for Phages (Supplementary Tables 3, 4). The top 30 unique contigs with the most reads abundance of DNA virus were assigned to families Circoviridae, Parvoviridae and Herpesviridae, meanwhile RNA virus were assigned to families Astroviridae, Coronaviridae, and Picornaviridae (Figure 1).

### Insect, Plant and Phage Viruses

**Figure 2:**
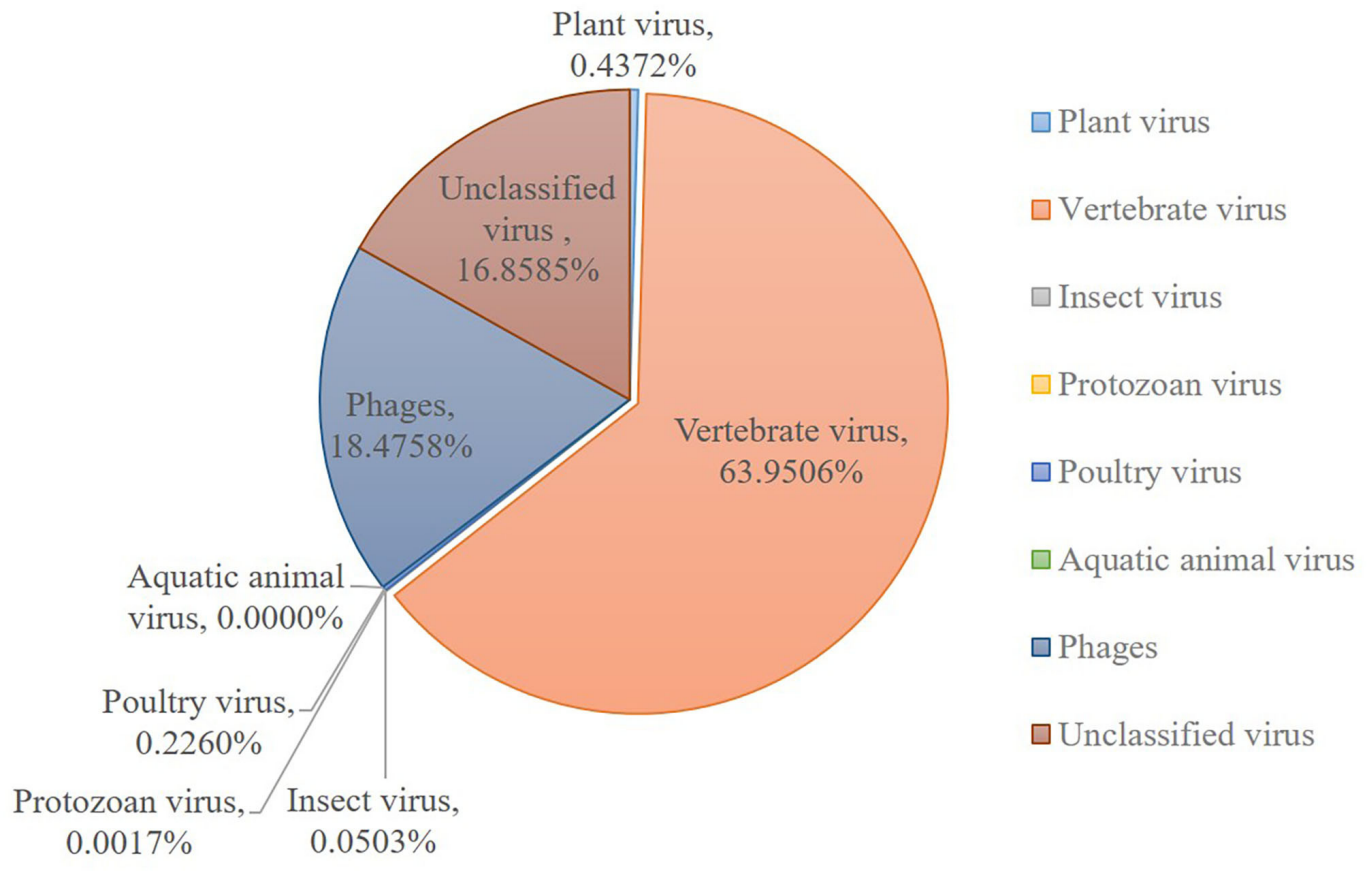
Host wise distribution of eukaryotic viruses cataloged in the dog gut virome. Summary of the sequence classification at different taxonomic ranks by their assigned homology results of host information.

The sequences mapping to insect and plant viruses comprised a proportion within the eukaryotic family viruses in the dog. Several dog virome sequences were homologous with the insect virus families of Polydnaviridae, Iridoviridae, Nimaviridae and Dicistroviridae, and plant virus families of Virgaviridae, Solemoviridae, Alphaflexiviridae and Bromoviridae, reflecting the diet and habitation of domestic dogs. The phages of the Microviridae, Siphoviridae, Ackermannviridae, Podoviridae, Myoviridae families, and unclassified phages (Figure 2).

### Vertebrate Viruses

**Figure 3:**
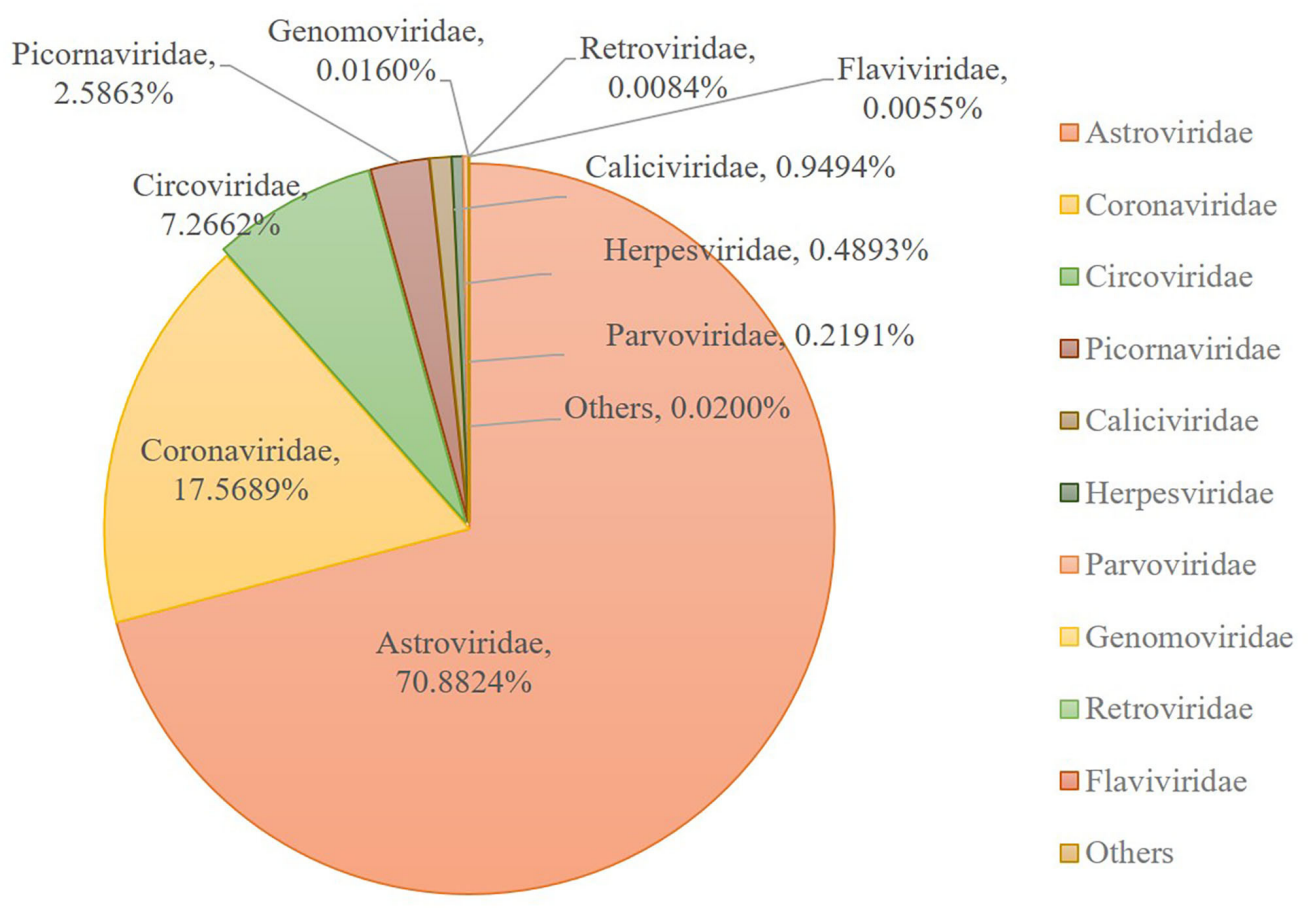
Taxonomic distributions of the eukaryotic virus-related sequences from dog gut virome. The number of sequences with identities to eukaryotic viruses was shown.

**Figure 4:**
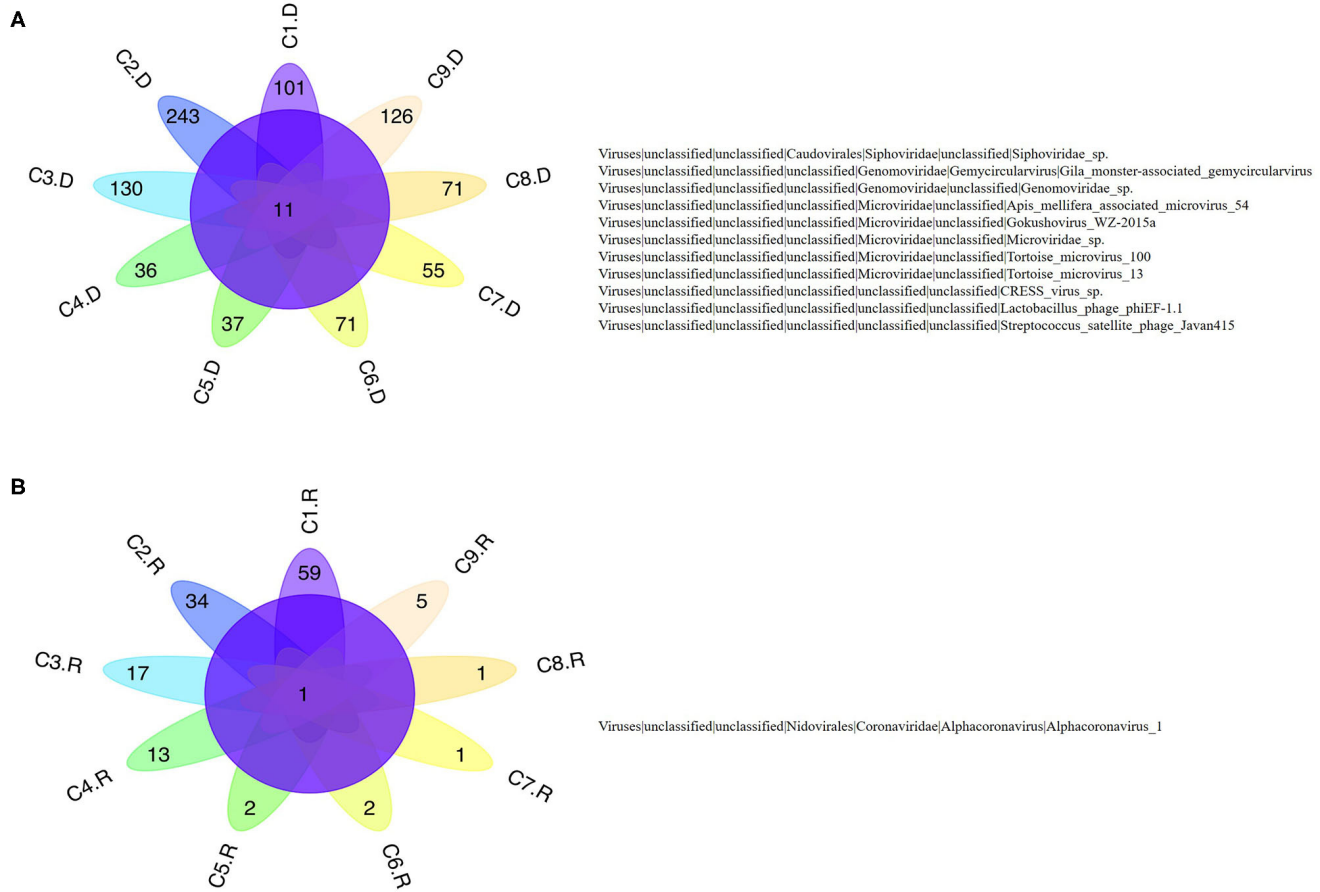
The shared virus distributions of the nine databases. **(A)** The shared virus distributions of DNA virome. **(B)** The shared virus distributions of RNA virome.

**Figure 5:**
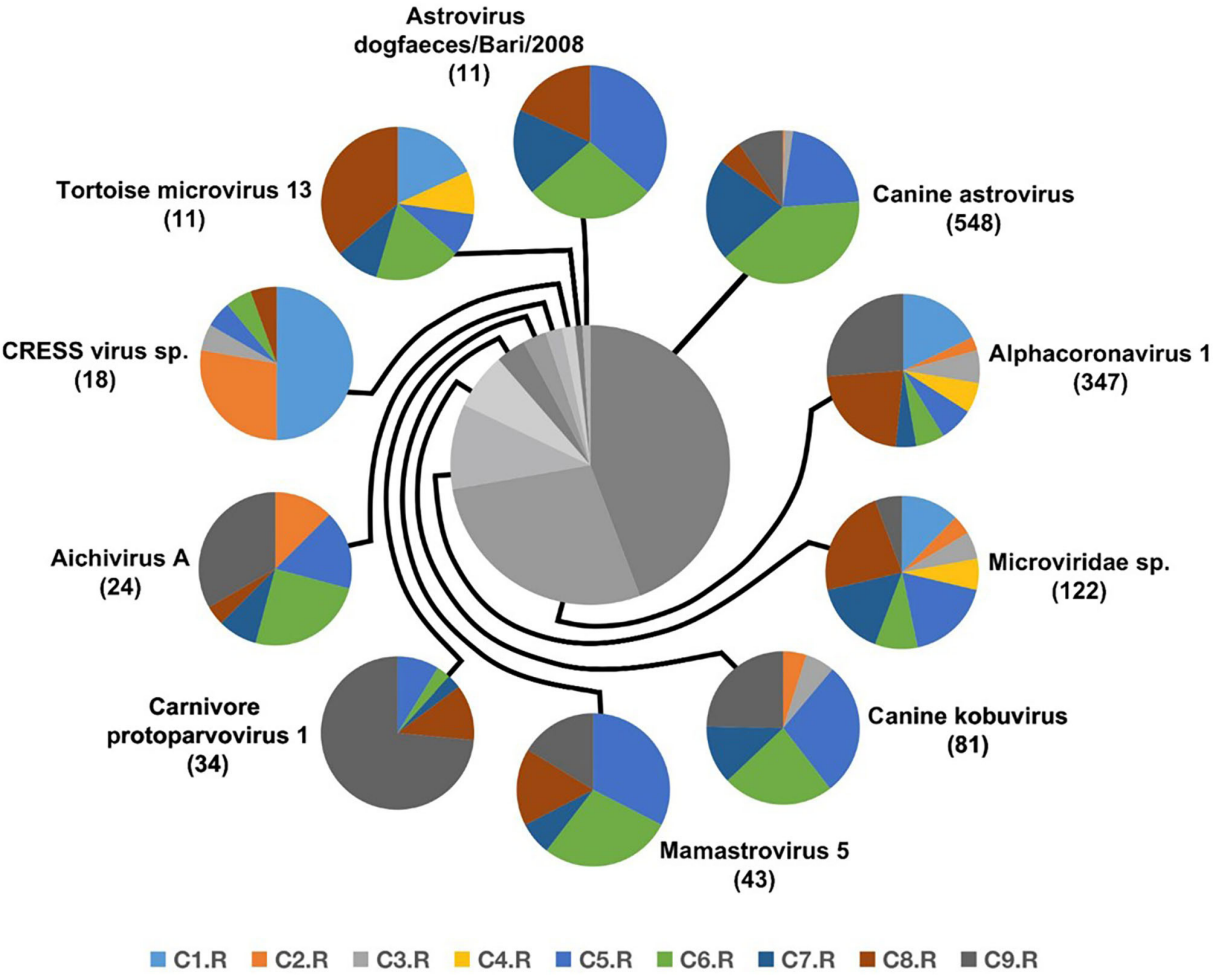
The composition of eukaryotic RNA viruses detected in dog gut virome. The center pie chart indicated the approximate percentages of the ten virus groups detected in all nine databases. The circumjacent smaller pie charts showed the approximate percentage of virus sequence from different databases. The databases are shown in different colors.

Numerous dog virome sequences had homology with the animal virus of the Astroviridae, Coronaviridae, Circoviridae, Picornaviridae, Caliciviridae, Herpesviridae, Parvoviridae, Genomoviridae, Retroviridae and Flaviviridae families (Figure 3). The shared viruses of all the nine databases were analyzed to investigate the frequency of different viruses. A total of 11 DNA viruses (Figure 4A) and one RNA virus (Figure 4B) were discovered in all samples. The 11 DNA shared viruses were phages except for the CRESS viruses. Significantly, the unique shared RNA virus was coronavirus. What's more, further abundance analysis of the animal virus in all the samples indicated that Alphacoronavirus was discovered in all the nine databases and with the secondary proportion. Although the canine astrovirus occupied the most proportion, only seven of nine databases were detected (Figure 5). Therefore, the relationship of different coronaviruses was further analyzed.

### Coronavirus

**Figure 6:**
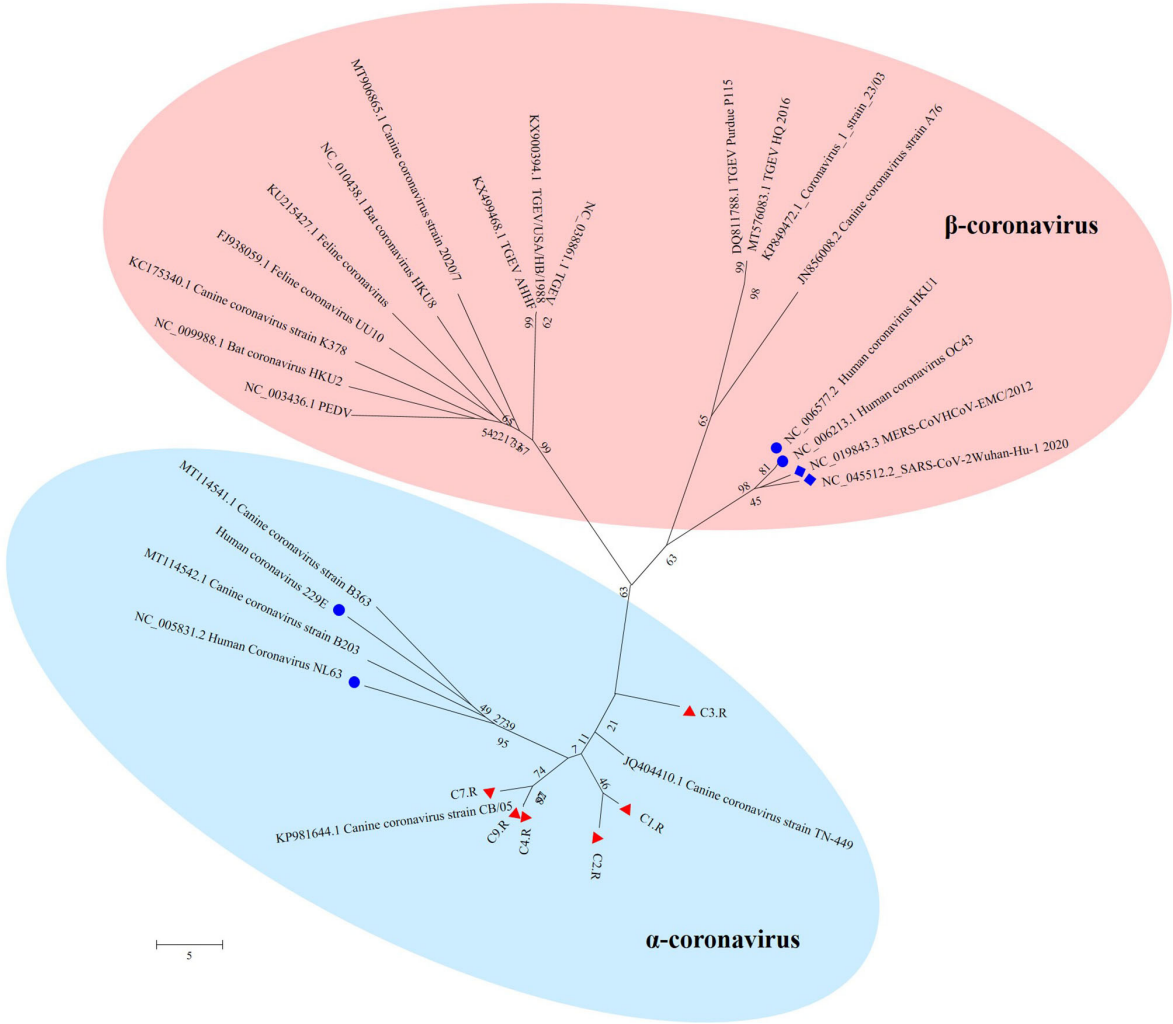
The phylogenetic tree of Coronavirus. The phylogenetic tree was generated by using the MEGA6 software version 6.06 with neighbor-joining method and bootstrapped with 1000 replicates. The assembled sequences in the study are labeled in red triangle. The labeled blue circle represents the Coronavirus from human, and the labeled blue diamond represents the Coronavirus from human resulting in pandemic.

The unique virus family confirmed in all the nine databases was Coronaviridae. Since none of the sampled dogs was vaccinated with coronavirus, the results could reflect the real Coronaviridae presence in the gut. Larger contigs were produced to assemble several coronavirus sequences. Whole genome sequences of strains belonging to Alphacoronavirus and Betacoronavirus genera were downloaded from the NCBI database. Together with the 6 sequences confirmed as Coronavirus in the study, all the reference sequences were aligned utilizing MEGA version 6.06 (38). CLUSTALW was used to align the sequence with the default settings (37). Further, the aligned sequences were trimmed to match the whole viral genomic obtained in the study. By means of MEGA version 6.06 (38), a phylogenetic tree was generated using the neighbor-joining method with 1,000 bootstrap resamples of the alignment data sets. As a result, the phylogenetic analysis showed Coronavirus contigs in the study clustered in Alphacoronavirus genera and these strains were divided into three sublineages (Figure 6). They showed a close relationship with canine coronavirus circulating in China, USA and Italy. C1.R and C2.R were clustered in the same sublineage, while C4.R, C7.R and C9.R were grouped into a group, which was closely related to canine coronavirus strain CB/05 circulating in Italy. C3.R showed a relatively distinct relationship with other viruses (Figure 6). The host range is largely determined by the coronaviral spike protein, which initiates cellular infection by promoting fusion of the viral and host cell membranes (40, 41). Although the viruses in the study clustered in different lineage with the human pandemic viruses, such as MERS and SARS-Cov, it is possible that they might eventually gain efficient transmissibility through the accumulation of mutations and reassortment especially within the spike gene. Significantly, the canine coronavirus B203 and B363 strains had a close relationship with the human coronavirus 229E and NL63 strains, indicating the potential risk of canine coronavirus to infect humans by obtaining the ability of cross-species transmission.

## Discussion

This research described the virome in fecal samples from 45 healthy dogs in metagenomics analysis by using next generation sequencing (NGS) technique. Except for the current research, there were only two previous shotgun metagenomic studies similarly investigating the fecal virome of dogs with diarrhea (42, 43). Comparing to the various investigations of the virome in numerous animal species and in different sites of the host body, the fecal virome of healthy domestic dogs has not been separately investigated, which will be more persuasive for the safety precaution. Our study successfully identified various viruses from 45 fecal samples from healthy domestic dogs. The identified viral sequences ranged from RNA and DNA families to known pathogens which were implicated in enteric disease. A total of 31 DNA and 32 RNA families of viruses were identified. Numerous dog virome sequences were homologous to the animal virus of the Astroviridae, Coronaviridae, Circoviridae, Picornaviridae, Caliciviridae, Herpesviridae, Parvoviridae, Genomoviridae, Retroviridae and Flaviviridae families; and phages of the Microviridae, Siphoviridae, Ackermannviridae, Podoviridae, Myoviridae families and unclassified phages. Further, several dog virome sequences had homologous with the insect viruses of the Polydnaviridae, Iridoviridae, Nimaviridae and Dicistroviridae families; and plant viruses of the Virgaviridae, Solemoviridae, Alphaflexiviridae and Bromoviridae families, which reflects the diet and habitation of dogs. These results show a high detection frequency of virus in extensively contacted animals and provide the associated viral genomes for further study or diagnose of corresponding diseases. In detail, the 63.95% of canine feces containing vertebrate viruses was consistent with the previous report (43).

The bacteriophages were the most common viral contigs identified from human and other animals fecal virome, especially from the samples with diarrhea (22, 44–47). Bacteriophages modify the diversity of bacterial populations due to their lytic life cycle (48). This conferred an advantage over bacterial species in the environmental niche (49). For example, a higher amount of bacteriophages that identified in the dogs with acute diarrhea to healthy dogs, might contribute to the bacterial dysbiosis (43). However, the identified bacteriophages in our study occupied 18.48%, following with the vertebrate viruses. The virome was analyzed based on healthy domestic dogs, which might possess a moderate amount of bacteriophages. Bacteriophages belonging to families Siphoviridae, Ackermannviridae, Podoviridae, Myoviridae and ssDNA family Microviridae were identified. However, this study did not assess the bacterial microbiome and yet analyzed contigs matching specific bacteriophages, therefore, a cross analysis of microbiome/virome is further necessary to elucidate interactions of bacteria and bacteriophages in dogs.

Among the viruses identified in this study, astrovirus was detected mainly in puppies with diarrhea and occasionally in healthy dogs in previous research (50–54). And the complete genome of two canine astroviruses was firstly described in the UK in 2015 (53). To date, canine astrovirus has been reported in Australia (43), USA (6), China (51), Italy (50, 55, 56), UK (53), France (52), Brazil (57), Korea (58) and Japan (54). Our metagenomic sequence data indicated that the most frequent vertebrate viral family in healthy dog samples was Astroviridae (70.88%), indicating Astroviridae has emerged with more frequency as time goes by.

To investigate the frequency of different viruses, the shared viruses of all the nine databases were analyzed. Significantly, Alphacoronavirus was identified in all the nine databases. Coronavirus is responsible for a variety of severe diseases including gastroenteritis and respiratory tract diseases. This virus has a wide range of hosts, such as horses, mice, rats, turkeys, chickens, swine, rabbits, cattle, dogs, cats and humans (59). Infections caused by coronavirus were identified in both animals and humans (60). The coronavirus can be shed in feces for up to 156 days in dogs (16, 17), which will increase the risk to infect other animals and even humans. The first human coronaviruses were identified in the 1960s from patients with the common cold. Since then, more and more viruses from this family were discovered. For example, the two pathogens causing severe acute respiratory syndrome (SARS) and the Middle East respiratory syndrome (MERS) (61), fatal respiratory disease in humans (62), were all from the Coronaviridae family. The severe acute respiratory syndrome coronavirus 2 (SARS-CoV-2) pandemic occurred in Wuhan in December 2019 represents a global threat (63). The primary entry receptor for SARS-CoV-2 is the Angiotensin-converting enzyme 2 (ACE2). A recent study indicated that the ACE2 of dogs facilitated SARS-CoV-2 entry into nonsusceptible cells (64). Significantly, SARS-CoV-2 was detected from two dogs which lived with the diagnosed humans, while the dogs remained asymptomatic during quarantine (65). All the evidence suggests that these are transmissions of SARS-CoV-2 across species (41). Classically, CCoV was considered to cause only self-limiting enteritis with mild diarrheal disease (66). However, the increase in disease severity in dogs and the emergence of novel CCoVs can be attributed to the high level of recombination within the spike gene that can occur during infection by more than one CCoV type in the same host (67). Therefore, there is the likelihood of continued emergence of novel CCoVs with distinct pathogenic properties in the future. Although the viruses in the study clustered in different lineage with the human pandemic viruses, such as MERS and SARS-Cov, it is possible that they might eventually gain efficient transmissibility through the accumulation of mutations and reassortment especially within the spike gene. Significantly, the canine coronavirus B203 and B363 strains in the phylogenetic tree had a close relationship with the human coronavirus 229E and NL63 strains, indicating the potential risk of canine coronavirus to infect humans by obtaining the ability of cross-species transmission. Therefore, it is urgent to further investigate the mechanism of mutation and reassortment of canine coronavirus in order to prepare for potential pandemic.

The viral metagenomic analyses are useful for the investigation of viral populations in the feces of healthy domestic dogs, which is significant in elucidating the epidemiological and biological role of companion animals in public health.

## Data Availability Statement

The data presented in the study are deposited in the SRA database, accession number (PRJNA734701).

## Ethics Statement

The animal study was reviewed and approved by The Animal Care and Use Committee of Shanghai Academy of Agricultural Science. Written informed consent was obtained from the owners for the participation of their animals in this study.

## Author Contributions

HL designed the research. YS, BL, JT, JC, and XS performed the research. YS analyzed data and wrote the paper. All authors contributed to the article and approved the submitted version.

## Conflict of Interest

The authors declare that the research was conducted in the absence of any commercial or financial relationships that could be construed as a potential conflict of interest.
