# Supplementary material for: The Gut Viral Metagenome Analysis of Domestic Dogs Captures Snapshot of Viral Diversity and Potential Risk of Coronavirus
Source: Front Vet Sci. 2021 Jul 7;8:695088. doi: 10.3389/fvets.2021.695088 (PMC8292670; doi:10.3389/fvets.2021.695088)
Supplement: Supplementary file 1 [file Table_1.docx]

**Supplementary material**

| Data ID | Raw Reads(PE) | Number of Reads Remaining after Filtering (%) | | | Assembly Data on Filtered Reads | | | | |
| --- | --- | --- | --- | --- | --- | --- | --- | --- | --- |
|  |  | Clean reads(PE) | Rm. Host Clean (PE) | Virus Reads (PE) | Total No. | Max Len. | Min Len. | N50 | GC (%) |
| C1.D | 59772059 | 31869248(53.32) | 31868774(100) | 261021(81.90) | 21920 | 102527 | 300 | 2089 | 40.69 |
| C2.D | 65972686 | 38665301(58.61) | 38643429(99) | 1674236(4.33) | 31892 | 104472 | 300 | 1993 | 48.47 |
| C3.D | 54180754 | 30909302(57.05) | 30908921(100) | 118871(0.38) | 18953 | 90493 | 300 | 1297 | 45.33 |
| C4.D | 52895719 | 31821493(60.16) | 31821102(100) | 67465(0.21) | 23849 | 181469 | 300 | 2071 | 41.75 |
| C5.D | 38518886 | 15931515(41.36) | 15897329(99.79) | 57489(0.36) | 14800 | 391609 | 300 | 2374 | 56.21 |
| C6.D | 39932592 | 22040588(55.19) | 22001166(99.82) | 65727(0.30) | 28012 | 643829 | 300 | 2957 | 54.91 |
| C7.D | 37515071 | 17671306(47.1) | 17640316(99.82) | 776295(4.39) | 24727 | 627790 | 300 | 1527 | 56.01 |
| C8.D | 38280953 | 25530394(66.69) | 25498203(99.87) | 42745(0.17) | 32807 | 602244 | 300 | 3300 | 55.49 |
| C9.D | 38495750 | 32163601(83.55) | 32133863(99.97) | 808713(2.51) | 43268 | 643828 | 300 | 5951 | 55.00 |
| C1.R | 46142526 | 28898869(62.63) | 7645202(26.46) | 40911(14.16) | 9640 | 75519 | 300 | 576 | 47.86 |
| C2.R | 56653920 | 32621798(57.58) | 11246021(34.47) | 162560(49.83） | 6959 | 19576 | 300 | 585 | 50.05 |
| C3.R | 51626274 | 28347339(54.91) | 10229586(36.09) | 5805203(20.48) | 7337 | 23198 | 300 | 562 | 54.22 |
| C4.R | 55085077 | 31117711(56.49) | 5049829(16.23) | 16907(0.05) | 9341 | 22132 | 300 | 487 | 50.91 |
| C5.R | 38930441 | 29821077(76.6) | 14196227(47.60) | 4928851(16.53) | 13818 | 37192 | 300 | 612 | 55.86 |
| C6.R | 35743324 | 24162277(67.6) | 18505247(76.59) | 14398927(59.59) | 9066 | 14180 | 300 | 553 | 53.70 |
| C7.R | 40743639 | 24416915(59.93) | 21096776(86.40) | 16859924(69.05) | 5167 | 14434 | 300 | 505 | 51.63 |
| C8.R | 41840367 | 31572332(75.46) | 28653223(90.75) | 139320(0.44) | 10552 | 74864 | 300 | 841 | 55.81 |
| C9.R | 35883739 | 27641940(77.03) | 10573799(38.25) | 607673(2.20) | 13231 | 34487 | 300 | 676 | 56.98 |

**Table S1. Overview of reads and contig sequences.** Rm. Host clean: number and percentage of reads after removing host sequence; Virus reads: number of reads mapped to the virus database. Total NO.：number of contigs.

| Data ID | Total contigs num | Max_len | virus contigs | Max_len |
| --- | --- | --- | --- | --- |
| C1.D | 21920 | 102527 | 1335 | 71094 |
| C2.D | 31892 | 104472 | 1742 | 90420 |
| C3.D | 18953 | 90493 | 1288 | 57998 |
| C4.D | 23849 | 181469 | 1107 | 71094 |
| C5.D | 14800 | 391609 | 326 | 39806 |
| C6.D | 28012 | 643829 | 340 | 224793 |
| C7.D | 24727 | 627790 | 274 | 63022 |
| C8.D | 32807 | 602244 | 359 | 42341 |
| C9.D | 43268 | 643828 | 558 | 191088 |
| C1.R | 9640 | 75519 | 411 | 14728 |
| C2.R | 6959 | 19576 | 189 | 19576 |
| C3.R | 7337 | 23198 | 362 | 23198 |
| C4.R | 9341 | 22132 | 357 | 22132 |
| C5.R | 13818 | 37192 | 252 | 8152 |
| C6.R | 9066 | 14180 | 335 | 7280 |
| C7.R | 5167 | 14434 | 177 | 14434 |
| C8.R | 10552 | 74864 | 172 | 2965 |
| C9.R | 13231 | 34487 | 250 | 7611 |
| Total | **325339** |  | **9834** |  |

**Table S2. The total contigs and viral contigs.** A total of 9834 contigs were assigned for virus species, accounting for 3.02% of the total contigs.

| Data ID | Total confirmed virus contigs | DNA contigs | Phage contigs | suspected virus contigs | suspected DNA | suspected phages |
| --- | --- | --- | --- | --- | --- | --- |
| C1.D | 306 | 306 | 289 | 3997 | 3970 | 3802 |
| C2.D | 714 | 708 | 695 | 4211 | 4184 | 3881 |
| C3.D | 426 | 408 | 386 | 2788 | 2776 | 2257 |
| C4.D | 192 | 178 | 147 | 3233 | 3224 | 2432 |
| C5.D | 84 | 74 | 72 | 1151 | 1134 | 1030 |
| C6.D | 53 | 50 | 38 | 883 | 873 | 759 |
| C7.D | 31 | 26 | 21 | 972 | 954 | 846 |
| C8.D | 43 | 39 | 36 | 954 | 934 | 830 |
| C9.D | 154 | 145 | 135 | 2874 | 2832 | 2500 |

**Table S3. The confirmed and suspected DNA virus contigs and phage contigs.**

| Data ID | Total confirmed virus contigs | RNA contigs | Phages contigs | suspected virus contigs | suspected RNA | suspected phages |
| --- | --- | --- | --- | --- | --- | --- |
| C1.R | 53 | 44 | 8 | 3516 | 144 | 2548 |
| C2.R | 24 | 17 | 4 | 1528 | 95 | 1031 |
| C3.R | 27 | 8 | 9 | 1792 | 90 | 1088 |
| C4.R | 47 | 30 | 6 | 4729 | 102 | 3178 |
| C5.R | 50 | 39 | 6 | 3224 | 327 | 2136 |
| C6.R | 47 | 34 | 9 | 1844 | 499 | 1014 |
| C7.R | 27 | 18 | 3 | 1205 | 315 | 662 |
| C8.R | 37 | 27 | 3 | 890 | 152 | 573 |
| C9.R | 68 | 56 | 0 | 2568 | 228 | 1678 |

**Table S4. The confirmed and suspected RNA virus contigs and phage contigs.**
